# Caffeic acid phenethyl amide ameliorates ischemia/reperfusion injury and cardiac dysfunction in streptozotocin-induced diabetic rats

**DOI:** 10.1186/1475-2840-13-98

**Published:** 2014-06-12

**Authors:** Yi-Jin Ho, An-Sheng Lee, Wen-Pin Chen, Wei-Lung Chang, Ying-Kang Tsai, Hsi-Lin Chiu, Yueh-Hsiung Kuo, Ming-Jai Su

**Affiliations:** 1 Department of Pharmacology, College of Medicine, National Taiwan University, 11F, No. 1, Sec. 1, Jen-Ai Road, Taipei 10051, Taiwan; 2 Department of Medicine, Mackay Medical College, New Taipei 252, Taiwan; 3 Department of Chemistry, National Taiwan University, Taipei 100, Taiwan; 4 Department of Chinese Pharmaceutical Sciences and Chinese Medicine Resources, China Medical University, Taichung 404, Taiwan; 5 Department of Biotechnology, Asia University, Taichung 413, Taiwan

**Keywords:** Diabetes, Ischemia/reperfusion injury, Caffeic acid phenethyl amide

## Abstract

**Background:**

Caffeic acid phenethyl ester (CAPE) has been shown to protect the heart against ischemia/reperfusion (I/R) injury by various mechanisms including its antioxidant effect. In this study, we evaluated the protective effects of a CAPE analog with more structural stability in plasma, caffeic acid phenethyl amide (CAPA), on I/R injury in streptozotocin (STZ)-induced type 1 diabetic rats.

**Methods:**

Type 1 diabetes mellitus was induced in Sprague–Dawley rats by a single intravenous injection of 60 mg/kg STZ. To produce the I/R injury, the left anterior descending coronary artery was occluded for 45 minutes, followed by 2 hours of reperfusion. CAPA was pretreated intraperitoneally 30 minutes before reperfusion. An analog devoid of the antioxidant property of CAPA, dimethoxyl CAPA (dmCAPA), and a nitric oxide synthase (NOS) inhibitor (Nω-nitro-l-arginine methyl ester [l-NAME]) were used to evaluate the mechanism involved in the reduction of the infarct size following CAPA-treatment. Finally, the cardioprotective effect of chronic treatment of CAPA was analyzed in diabetic rats.

**Results:**

Compared to the control group, CAPA administration (3 and 15 mg/kg) significantly reduced the myocardial infarct size after I/R, while dmCAPA (15 mg/kg) had no cardioprotective effect. Interestingly, pretreatment with a NOS inhibitor, (l-NAME, 3 mg/kg) eliminated the effect of CAPA on myocardial infarction. Additionally, a 4-week CAPA treatment (1 mg/kg, orally, once daily) started 4 weeks after STZ-induction could effectively decrease the infarct size and ameliorate the cardiac dysfunction by pressure-volume loop analysis in STZ-induced diabetic animals.

**Conclusions:**

CAPA, which is structurally similar to CAPE, exerts cardioprotective activity in I/R injury through its antioxidant property and by preserving nitric oxide levels. On the other hand, chronic CAPA treatment could also ameliorate cardiac dysfunction in diabetic animals.

## Background

Numerous clinical studies have shown that subjects with diabetes face an increased risk of mortality from coronary heart disease, such as myocardial infarction and stroke [1]. Accordingly, the infarct size of the heart is increased in streptozotocin (STZ)-induced type 1 diabetic rats under ischemia/reperfusion (I/R) injury [2,3].

In the pathology of myocardial infarction, injury is more dominant from reperfusion than that from ischemia and reactive oxygen species (ROS) generation is thought to be the main cause of reperfusion injury [4]. Therefore, antioxidant therapy should effectively reduce I/R injury [5].

Caffeic acid phenethyl ester (CAPE) is the major component in *propolis* extracts with known anti-inflammatory [6], anti-viral [7], cancer cell inhibitory [8], anti-bacterial [9], antioxidant [10], and free radical scavenging activities [9]. CAPE significantly decreased fasting blood glucose, alanine aminotransferase, cholesterol, and triglyceride levels and protected the brain against oxidative stress and inflammation in diabetic rats [11,12]. The 12-week oral administration of CAPE (30 mg/kg) slowed the atherosclerosis progress in apolipoprotein E-deficient mice [13]. In addition, CAPE administration protects many organs such as the brain [14], bone marrow [14,15], kidney [16], lung [17] and ovary [18] against I/R injury. In the heart, CAPE can also protect against I/R injury by various mechanisms [19-23] including its antioxidant activity.

A CAPE analog, caffeic acid phenethyl amide (CAPA, *N-*trans-caffeoyl-β-phenethylamine), synthesized from 3,4-methylene-dioxy-cinnamic acid, with an amide linkage between caffeic acid and the phenethyl group that resists hydrolysis within the circulation, was recently found to be more stable than CAPE in rat plasma [24]. CAPA presents a significantly longer elimination half-life in the systemic circulation than CAPE after intravenous administration into male rats [25]. It exerts beneficial effects by its free radical scavenging and antioxidant activity [26,27] and displays a cytoprotective effect against H_2_O_2_-induced cell death in human umbilical vascular endothelial cells [28]. CAPA could also improve glucose homeostasis by α-glucosidase inhibition [29], adiponectin induction [30]*in vitro*, stimulating insulin secretion and reducing plasma glucose in diabetic rats and mice [31,32]. It has also been shown to attenuate the progression of vascular dysfunction in diabetic rats [31], protect hearts against diet- and STZ- induced metabolic changes and decrease infarct size after global I/R by increasing coronary flow [33], and mitigate cardiac dysfunction in abdominal aortic banding-induced ventricular hypertrophy [34], indicating that CAPA may be beneficial in the treatment of diabetes and its cardiovascular complications.

The aim of this study was to characterize the effects of CAPA on I/R injury in normal and type 1 diabetic rats.

## Research design and methods

### Chemicals

**Figure 1:**
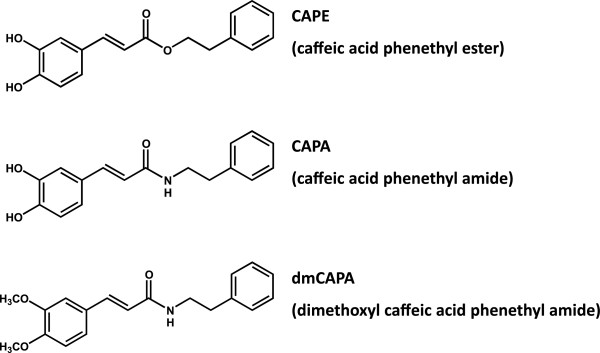
Chemical structures of CAPE, CAPA and dmCAPA.

Sodium pentobarbital, STZ, Nω-nitro-l-arginine methyl ester (l-NAME), thiobutabarbital sodium salt hydrate (Inactin® salt hydrate), methylene blue, 2,3,5-triphenyltetrazolium chloride (TTC), and poly-ethylene glycol 400 (PEG400) were purchased from Sigma-Aldrich, MO, USA. The chemicals for the physiological buffer were purchased from J.T. Baker, Capital Scientific Inc. and Wako Pure Chemical Industries. CAPA and 3,4-dimethoxyl caffeic acid phenethyl amide (dmCAPA, Figure 1) were synthesized and obtained from Institute of Chemistry, National Taiwan University [31]. CAPA was dissolved in PEG400 30% for intraperitoneal injection or suspended in distilled water for oral administration.

### Animals and the induction of diabetic rats

**Figure 2:**
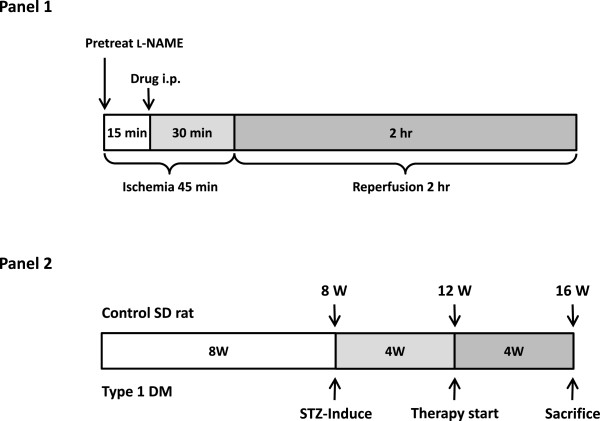
**Ischemia/reperfusion model and chronic treatment time course in type 1 diabetic rats.** All animals underwent coronary artery occlusion for 45 min followed by 2 hours of reperfusion. CAPA (3 and 15 mg/kg) and dmCAPA (15 mg/kg) were administered intraperitoneally 30 min before reperfusion while the NOS inhibitor (l-NAME; 3 mg/kg, intraperitoneal) was given before LAD occlusion **(panel 1)**. For chronic treatment, type 1 diabetes was induced by STZ over 4 weeks in 8-week-old rats that were then treated with vehicle or CAPA for 4 weeks **(panel 2)**.

Eight-week-old male Sprague–Dawley rats (BioLASCO Taiwan, Co., Ltd, Taipei, Taiwan) weighing 250–300 g (bred in a Lab Animal Center, National Taiwan University, Taiwan) were used. The animals were housed in a conditioned environment (22 ± 1°C, 55 ± 5% relative humidity, 12-h light and darkness cycle, free access to food and water). Throughout the studies, all efforts were made to minimize animal pain and suffering. All animal procedures were performed according to the *Guide for the Care and Use of Laboratory Animals* of the National Institutes of Health, as well as the guidelines of the Animal Welfare Act, and the animal studies were approved by the Institutional Animal Care and Use Committee of the College of Medicine, National Taiwan University (certificate no. 20110073).To evaluate the effects of CAPA on infarct size in healthy rats, the left anterior descending coronary artery (LAD) of 8-week-old rats was occluded for 45 min and reperfused for 2 hours; CAPA and dmCAPA were given intraperitoneally 30 min before reperfusion, while the nitric oxide synthase (NOS) inhibitor was given 15 min before CAPA and dmCAPA administration (Figure 2, panel 1).

For the induction of diabetes, fasting rats were anesthetized with sodium pentobarbital (30 mg/kg) and intravenously injected with STZ (60 mg/kg freshly dissolved in sterile, non-pyrogenic 0.9% NaCl solution in a volume of 1 mL/kg body weight [35]) through the tail vein after a 72-h fast [36]. Two weeks after the STZ injection, the animals were considered to have type 1 diabetes if the plasma glucose level was > 350 mg/dL and diabetic features such as polyuria, polydipsia, and hyperphagia were observed [37].Four weeks after the STZ induction, the animals were divided into three groups: age-matched non-diabetic control animals; STZ-diabetic rats administered vehicle (distilled water) for 4 weeks; and, STZ-diabetic rats administered CAPA (1 mg/kg/day) for 4 weeks (Figure 2, panel 2).

### Surgical procedure of I/R injury in rat heart

Rats underwent myocardial ischemia by the temporary occlusion of the LAD close to its origin to induce I/R injury as previously described [38]. Briefly, the rats were intraperitoneally anesthetized with Inactin® hydrate (80 mg/kg) and urethane (4 g/kg) [39] on an operating table equipped with a heater to maintain the proper temperature. After undergoing a tracheotomy, the animals were ventilated with room air by a rodent ventilator (Model 683, Harvard Apparatus, South Natick, MA, USA) with a stroke volume of 10 mL/kg body weight at a rate of 65 strokes/min. The chest was opened and the ribs were gently spread. The heart was quickly expressed out of the thoracic cavity, and a 7/0 silk ligature was placed under the LAD. The heart was repositioned in the chest and the animal was allowed to recover for 20 min. A small plastic snare formed from a polyethylene tubing Portex P-270 cannula was threaded through the ligature and placed in contact with the heart. The ligature was tightened to occlude the artery and reperfusion was initiated by withdrawal of the polyethylene tubing. Regional myocardial ischemia was verified by the presence of a cyanotic zone in the area of distribution of the occluded vessel and by changes in the electrocardiogram consistent with the presence of transmural regional myocardial ischemia (ST-segment elevation).

### Estimation of myocardial damage

At the end of the experiment, the ischemic and perfused areas of heart were determined by injection of methylene blue (2 mL; 0.2% in 0.9% NaCl) through the jugular vein after coronary artery re-occlusion. The rat was sacrificed and the heart was differentiated into perfused (blue) and occluded regions. The occluded region (defined as the "area at risk" [AAR]) was cut out, weighed, and expressed as a percentage of ventricle weight. Thereafter, the occluded tissue was sliced into l-mm sections for incubation in TTC (in saline, 1%) at 37°C for 30 min. The sections were placed in 10% formaldehyde in saline for 2 days. The infarcted (white) tissue was excised and weighed. Infarct size was expressed as a percentage of weight in the occluded zone [40].

### Tissue malondialdehyde (MDA) content analysis

The unperfused zone of cardiac tissue was collected as described above for the determination of MDA content using a commercial kit from Sigma-Aldrich Co. LLC (Catalog Number MAK085). Briefly, tissue samples were homogenized in phosphate-buffered saline solution and then completely homogenized by ultra-sonication (40 V for 15 s). The supernatants were collected in glass tubes and reacted with sodium acetate solution containing thiobarbituric acid (TBA) at 95°C for 60 min. After centrifugation, the supernatants were collected and the resulting TBA-reactive substances were measured spectrophotometrically at 532 nm absorbance and expressed as MDA equivalents (nmol) per milligram of wet tissue as a measure of lipid peroxidation [41].

### Tissue myeloperoxidase (MPO) activity analysis

The occluded zone of cardiac tissue was collected as described above for the determination of MPO activity using a commercial kit from Sigma-Aldrich Co. LLC (Catalog Number MAK068). Briefly, tissue samples were homogenized in hexadecyl trimethyl ammonium bromide and dissolved in potassium phosphate. After centrifugation, the supernatants were collected and mixed with *o*-dianisidine hydrochloride and hydrogen peroxide in phosphate buffer. MPO activity was measured spectrophotometrically at 412 nm absorbance. MPO activity was defined as the quantity of enzyme degrading 1 mmol of peroxide per min at 37°C and was expressed in units per milligram of wet tissue as a measure of neutrophil activation [40].

### Physiological hemodynamic parameters recording

Polyethylene catheters (PE 50) were inserted into the common carotid artery of the rats to measure blood pressure and the three-lead electrodes were inserted subcutaneously to monitor the electrocardiography. The arterial blood pressure and electrocardiography data were continuously recorded by a PowerLab 4/30 data acquisition system (ADInstruments, Castle Hill, NSW, Australia), while the cardiac function was assessed by a pressure and volume microtip catheter (1.9 F; Scisense Instruments, Ontario, Canada). This catheter was inserted into the left ventricle to measure pressure and volume. The pressure was measured at a sampling rate of 1,000/s using a PONEMAH real-time acquisition interface P3P Plus coupled to an analog-to-digital converter (ACQ-16). All pressure volume (PV) loop data were analyzed using the PONEMAH Life Sciences Suite cardiac PV analysis program from Data Sciences International (St. Paul, MN, USA). The hemodynamic parameters included left ventricle end-systolic pressure (LVESP), LV end-diastolic pressure (LVEDP), stroke volume (SV), maximum positive value (+dP/dt), maximum negative value (-dP/dt) of the first derivative of the pressure, stroke work (SW), and ejection fraction. The preload-independent cardiac contractility parameters were also determined under conditions of changing preload, elicited by transient compression of the abdominal inferior vena cava. These measurements include end-systolic pressure volume relationship (ESPVR), end-diastolic pressure volume relationship (EDPVR), and the preload recruitable stroke work (PRSW). In addition, arterial volume elastance (Ea) was calculated as the ratio of LVEDP at the point in the cycle where the PV ratio peaks versus the stroke volume under different experimental conditions [42].

### Data analysis

Values are expressed as mean ± standard error of mean (SEM). The data were subjected to one-way analysis of variance followed by a multiple-comparison test (Bonferroni-test). Values of *P* < 0.05 were considered statistically significant.

## Results

### CAPA protected the heart from I/R injury via a nitric oxide (NO)-dependent pathway

**Figure 3:**
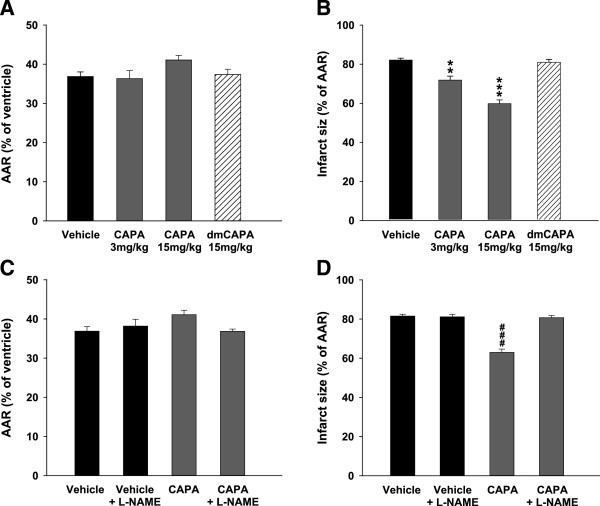
**Effects of CAPA, dmCAPA andl-NAME pretreatment on I/R injury. (A)** Area at risk (AAR; % of ventricle) and **(B)** infarct size (% of AAR) in control rats treated with vehicle and in rats treated with CAPA (3 or 15 mg/kg) and dmCAPA (15 mg/kg). **P < 0.01 and ***P < 0.001 compared to vehicle. **(C)** AAR (% of ventricle) and **(D)** infarct size (% of AAR) in control rats treated with vehicle and in rats pretreated with l-NAME (3 mg/kg) and treated with CAPA (15 mg/kg). ^###^*P* < 0.001 compared to vehicle. Data (mean ± SEM) were obtained from 6–8 animals.

To investigate the cardioprotective effects of CAPA against I/R injury *in vivo*, rats were intraperitoneally treated with CAPA (3 and 15 mg/kg) or dmCAPA (CAPA derivative, methylation at the hydroxyl groups, no antioxidant activity; 15 mg/kg; Figure 1) 30 min before the reperfusion. Regional myocardial I/R was established by ligating the LAD of the rat heart for 45 minutes followed by reperfusion for 2 hours *in vivo* (Figure 2, panel 1). Hearts were analyzed by TTC to quantify the infarct size in the AAR. The AAR did not differ significantly among any of the groups of hearts (Figure 3A). The administration of 3 mg/kg and 15 mg/kg CAPA dose-dependently reduced I/R-induced infarct size from 81.5% in control hearts to 70.2% (*P* < 0.01) and 62.9% (*P* < 0.001), respectively. However, dmCAPA had no effect on infarct size (Figure 3B).

On the other hand, pretreatment with the NOS inhibitor (l-NAME 3 mg/kg) could effectively abolish the effect of CAPA on infarct size without changing AAR (Figures 3C and D).

### CAPA decreased the MDA content and MPO activity in I/R injury

**Figure 4:**
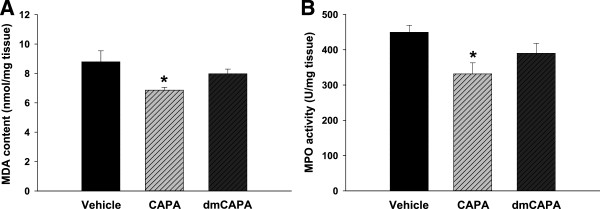
**Effects of CAPA treatment on MDA levels and MPO activity.** We administered CAPA (15 mg/kg, n = 10) and dmCAPA (15 mg/kg, n = 5) after 45 minutes of LAD ligation followed by 2 hours of reperfusion. After I/R, the tissue in the area at risk was collected for measurement of MDA levels **(A)** and MPO activity **(B)**. *P < 0.05 compared with controls.

We evaluated the effects of CAPA on lipid peroxidation and neutrophil activation by evaluating MDA content and MPO activity, respectively. CAPA administration (15 mg/kg) significantly reduced MDA content compared to vehicle (*P* < 0.05; Figure 4A), which means that CAPA could reduce the oxidative stress level in the heart during I/R. Moreover, CAPA could reduce MPO activity compared to vehicle (*P* < 0.05; Figure 4B). However, neither MDA content nor MPO activity significantly reduced with the same dosage of dmCAPA.

### The chronic effect of CAPA on body weight and heart weight in diabetic rats

**Figure 5:**
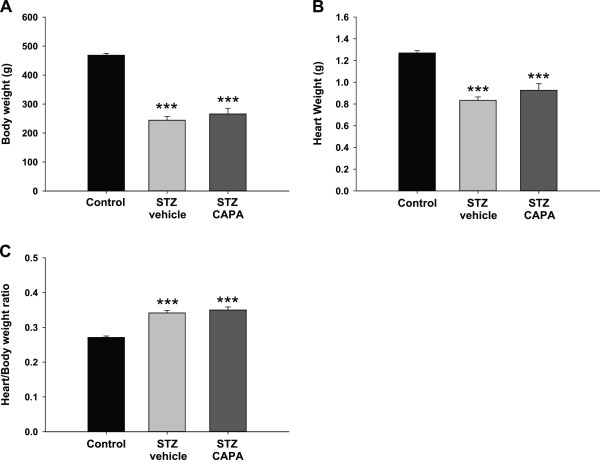
**Effects of CAPA treatment on body weight and heart weight.** After I/R, the body weight **(A)**, heart weight **(B)**, and heart/body weight ratio **(C)** were calculated. For chronic treatments, the animals were divided into three groups: control, age- and sex-matched normal rats, n = 16; STZ-vehicle, age- and sex-matched diabetic animals administered distilled water orally for 4 weeks starting 4 weeks after STZ induction, n = 13; and STZ-CAPA, CAPA 1 mg/kg administered orally once daily for 4 weeks starting 4 weeks after STZ induction, n = 6. Data are expressed as mean ± SEM. ***P < 0.001 compared with control group.

To investigate the effects of chronic CAPA treatment on cardiac function in diabetic rats, the STZ-induced type 1 diabetic rats were divided into two groups: one group treated with vehicle (distilled water) and a second group treated with CAPA (1 mg/kg, orally). After 4 weeks, both body weight and heart weight of vehicle-treated diabetic animals were lower than those of the age-matched animals (244.2 ± 12.8 g and 0.83 ± 0.03 g vs. 468.5 ± 6.1 g and 1.27 ± 0.02 g, *P* < 0.001; Figures 5A and B). CAPA treatment did not affect body weight or heart weight of diabetic animals (265.7 ± 19.7 g and 0.93 ± 0.06 g, respectively). However, the ratio of heart weight to body weight was higher in both diabetic groups treated with vehicle and CAPA (0.34 ± 0.03 and 0.35 ± 0.02, respectively) than in the control group (0.27 ± 0.01, *P* < 0.001; Figure 5C).

### CAPA increased heart rate and mean blood pressure during the I/R period in diabetic rats

**Figure 6:**
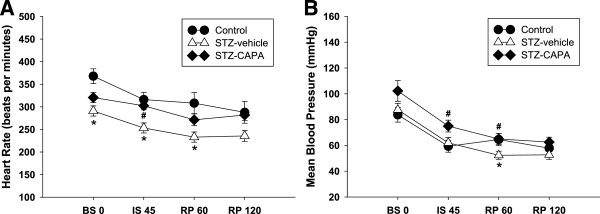
**Effects of CAPA treatment on heart rate and mean blood pressure in diabetic rats.** The heart rate **(A)** and mean blood pressure **(B)** during the I/R period in control and diabetic rats were recorded. For chronic treatments, the animals were divided into three groups: control, age- and sex-matched normal rats, n = 16; STZ-vehicle, age- and sex-matched diabetic animals administered distilled water orally for 4 weeks starting 4 weeks after STZ induction, n = 13; and STZ-CAPA, CAPA 1 mg/kg administered orally once daily for 4 weeks starting 4 weeks after STZ induction, n = 6. BS 0, basal value just before ischemia; IS 45, 45 min after ischemia but just before reperfusion; RP 60, 60 min after reperfusion; RP 120, 120 min after reperfusion. Data are expressed as mean ± SEM. *P < 0.05 compared with control group and ^#^*P* < 0.05 compared with STZ-vehicle group.

The mean heart rates of STZ-vehicle diabetic rats at the time before (BS 0), 45 min after ischemia (IS 45), and 60 min after reperfusion (RP 60) were significantly lower than those of age-matched control (290.9 ± 11.2 bpm, 253.2 ± 11.9 bpm, and 233.0 ± 11.3 bpm vs. 367.9 ± 16.3 bpm, 316.1 ± 16.2 bpm, and 308.2 ± 23.3 bpm, respectively; Figure 6A). CAPA treatment reversed the decrease in heart rate in diabetic rats, especially at the time point of IS 45 compared to the STZ-vehicle group (302.5 ± 9.1 bpm, *P* < 0.05). Similarly, the mean blood pressure was lower in STZ-vehicle diabetic rats than in control rats 60 min after reperfusion (52.3 ± 3.1 mmHg vs. 64.6 ± 4.7 mmHg), but CAPA could significantly ameliorate this effect (65.0 ± 4.2 mmHg, *P* < 0.05; Figure 6B).

### CAPA decreased the infarct size after I/R in diabetic rats

**Figure 7:**
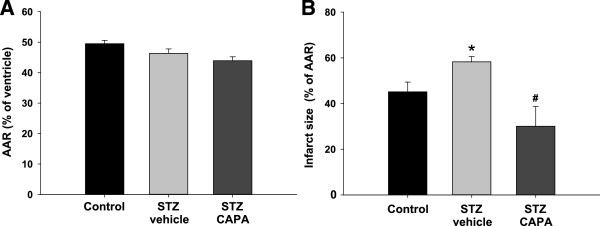
**Effects of CAPA treatment on infarct size in diabetic rats.** After I/R injury, area at risk **(A)** and infarct size/area at risk ratio **(B)** in diabetic rats were calculated. For chronic treatments, the animals were divided into three groups: control, age- and sex-matched normal rats, n = 16; STZ-vehicle, age- and sex-matched diabetic animals administered distilled water orally for 4 weeks starting 4 weeks after STZ induction, n = 13; and STZ-CAPA, CAPA 1 mg/kg administered orally once daily for 4 weeks starting 4 weeks after STZ induction, n = 6. Data are expressed as mean ± SEM. *P < 0.05 compared with control group and ^#^*P* < 0.05 compared with STZ-vehicle group. BS 0, basal value just before ischemia; IS 45, 45 min after ischemia but just before reperfusion; RP 60, 60 min after reperfusion; RP 120, 120 min after reperfusion.

Eight weeks after the STZ induction, the area at risk was similar among control, STZ-vehicle and STZ-CAPA diabetic rats (Figure 7A), while the infarct size was significantly increased in the STZ-vehicle diabetic animals (58.3 ± 2.3% vs. 45.1 ± 4.3% in age-matched normal rats, *P < 0.05). Chronic treatment of CAPA could decrease the infarct size to 30.1 ± 8.7% (^#^*P* < 0.05; Figure 7B).

### CAPA ameliorated I/R-induced cardiac dysfunction in diabetic rats on PV loop analysis

**Figure 8:**
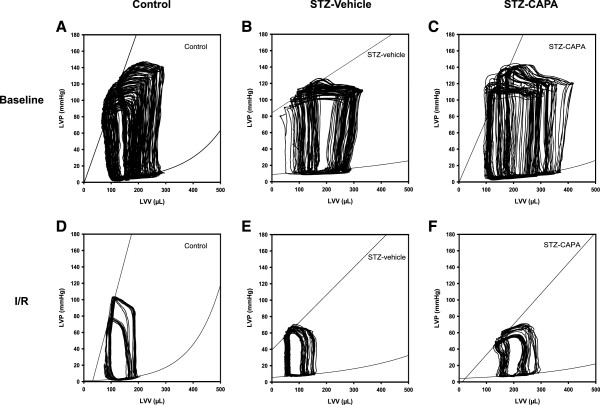
**Representative pressure–volume loops during preload reduction before and after I/R in diabetic rats.** The PV loops are derived from left ventricalar pressure (LVP) versus left ventricular volume (LVV) in the cardiac cycle diagram by transiently compressing the abdominal inferior vena cava before and after I/R. **A-C**, baseline PV loops before I/R; **D-F**, PV loops after I/R. Control (**A**, n = 16; and **D**, n = 6); diabetic rats administered vehicle (STZ-vehicle; **B**, n = 13; **E**, n = 6), diabetic rats administered CAPA (STZ-CAPA; **C**, n = 6; **F**, n = 5).

The cardiac functions of control, STZ-vehicle, and STZ-CAPA diabetic rats were measured by pressure-volume loops before and after I/R. The PV loops are derived from LV pressure versus LV volume in the cardiac cycle diagram by transiently compressing the abdominal inferior vena cava (Figure 8).

**Figure 9:**
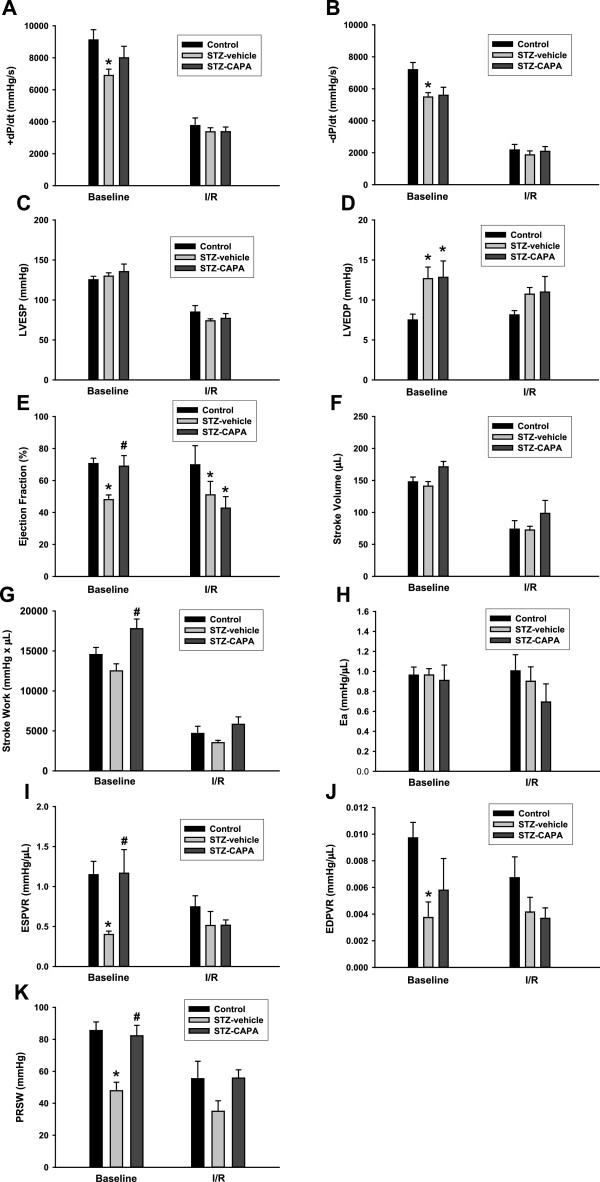
**PV loop analysis before and after I/R in diabetic rats.** The parameters of PV loop analysis are derived from LV pressure and LV volume. Ventricular contractility assessment is shown in maximum rising (+dP/dt, **A**) and falling (-dP/dt, **B**) velocity, left ventricular end systolic (LVESP, **C**) and end diastolic pressure (LVEDP, **D**) of the ventricular pressure that occurs during the cardiac cycle. **E**, ejection fraction (%). **F**, stroke volume. **G**, stroke work. Arterial elastance (Ea, **H**). End-systolic pressure volume relationship (ESPVR, **I**) and end-diastolic pressure volume relationship (EDPVR, **J**). Preload recruitable stroke work (PRSW, **K**). All values are represented as mean ± SEM before (baseline) and after I/R (I/R). *P < 0.05 compared to age- and sex-matched non-diabetic control rats. ^#^*P* < 0.05 versus age- and sex-matched diabetic rats treated with vehicle.

The maximum velocity of both contraction and relaxation in STZ-vehicle diabetic rats were significantly lower than those of age-matched control rats before I/R (6895.5 ± 393.9 mmHg/s and 5485.0 ± 276.6 mmHg/s vs. 9120.2 ± 634.5 mmHg/s and 7195.4 ± 450.1 mmHg/s, *P* < 0.05; Figures 9A and B). CAPA treatment did not reverse the decrease in the maximum rate of relaxation but seemed to have the activity to reverse the decrease in the maximum rate of contraction in STZ-diabetic rats. However, there were no significant differences among any of the groups after I/R.

In LVESP, there were no differences among any of the groups before or after I/R (Figure 9C), but the mean LVEDP values of the diabetic groups were higher than that of the control group at baseline (12.7 ± 1.4 and 12.8 ± 2.1 mmHg in the STZ-vehicle and STZ-CAPA groups compared to 7.5 ± 0.7 mmHg in the control group, *P* < 0.05; Figure 9D).

Figure 9E shows that the ejection fraction significantly decreased in STZ-vehicle diabetic rats before and after I/R compared to the control group (48.0 ± 3.0% and 51.0 ± 8.4% vs. 70.6 ± 3.4% and 69.8 ± 12.0%, respectively). CAPA treatment reversed the decrease before I/R (69.0 ± 6.6%, *P* < 0.05; Figure 9E), but not after reperfusion (42.8 ± 7.2%). There were no significant changes in stroke volume or stroke work among any of the groups before or after I/R except that STZ-CAPA diabetic rats had a higher mean stroke work value than the STZ-vehicle diabetic group (17785.4 ± 1206.4 vs. 12498.6 ± 902.3 mmHg × μL, *P* < 0.05; Figures 9F and G).In addition to the above parameters, PV loops at different preloads could be used to derive several preload-independent parameters. At baseline, ESPVR, EDPVR, and PRSW were lower in the STZ-vehicle group than in the control group (0.40 ± 0.04 mmHg/μL, 0.0037 ± 0.0012 mmHg/μL, and 47.8 ± 5.3 mmHg vs. 1.14 ± 0.40 mmHg/μL, 0.0097 ± 0.0012 mmHg/μL, and 85.5 ± 5.3 mmHg, respectively; Figures 9I, J, and K), but only ESPVR and PRSW were significantly reversed in the STZ-CAPA group (1.17 ± 0.30 mmHg/μL and 82.2 ± 6.5 mmHg).

## Discussion

### The antioxidant effect of CAPA on I/R injury

Myocardial infarction usually results from thrombus [43], vasospasm [44], routine coronary angioplasty [45], or open-heart operation [46]. Although restoration of blood flow is the only way to save the myocardium from ischemic injury, reperfusion can exacerbate the myocardial damage caused by ischemia. The mostly recognized cause of reperfusion injury is a burst of free radicals including hydrogen peroxide, hydroxyl radical, and superoxide radical that is formed during reperfusion and plays an important role in the pathophysiological mechanism of I/R injury [4]. Moreover, treatment with antioxidant remedies or agents capable of inducing antioxidant enzymes such as glutathione peroxidase and superoxide dismutase have a cardioprotective effect against I/R injury in experimental animals *in vivo* and *in vitro*[47]. Therefore, antioxidant therapy may be an effective process for treating myocardial infarction. However, predicting when myocardial infarction occurs and preventing the infarction with long-term antioxidants use is impractical, and administering medications after ischemia and before reperfusion is a more reasonable way to treat patients with myocardial infarction. Therefore, we administered CAPA 30 min prior to reperfusion to evaluate the cardiac effect of CAPA in I/R. The 2,2-diphenyl-1-picrylhydrazyl radical scavenging activity of CAPA, as shown by an EC_50_ of 18.6 ± 3.2 μM (comparable to the EC_50_ of 15.6 ± 2.0 μM CAPE, data not shown), was in accordance with that seen in other studies of the structure action relationship of CAPE and CAPA [26,48] and may reduce tissue oxidative stress and increase tissue availability of NO. In addition, our studies in type 2 diabetic mice have shown that CAPA treatment could increase manganese superoxide dismutase in fat tissue [33], but whether this effect is involved in improving the hemodynamic function of STZ-induced diabetic rats remains unknown.

CAPE administration protected the heart from I/R injury with reduced levels of oxidative stress such as MDA [21]. In our study, CAPA administration reduced MDA to a level comparable to that seen in the control group. An analog of CAPA, dmCAPA, which has no radical-scavenging activity, exhibited no cardioprotective effect on infarct size, suggesting that CAPA, like CAPE, exerts a cardioprotective effect mainly through its antioxidant property. Compared with vehicle treatment, the MPO activity reduced with CAPA administration, but not with dmCAPA. This finding suggests that the cardioprotective effect of CAPA could be partly attributed to the reduced inflammatory response by its antioxidant ability.

### NO preservation effect of CAPA on I/R injury

Although the role of NO remains controversial, myocardial I/R injury is exacerbated in the absence of endothelial cell NOS [49]; and endothelial NOS is able to regulate vascular tension [50], inhibit platelet aggregation [51], scavenge ROS, and stimulate endothelial regeneration to protect the heart and blood vessels [52]. CAPA inhibited lipopolysaccharide/interferon-γ (LPS/IFN-γ)-induced inducible NOS (iNOS) expression and NO production [53], while a CAPA-induced increase in coronary blood flow was prevented by a NOS inhibitor, which suggests that CAPA may enhance coronary blood flow by increasing NO availability or level [31]. In our study, pretreatment with the NOS inhibitor l-NAME eliminated the cardioprotective effect of CAPA on infarct size reduction, suggesting that NO is an important factor responsible for cardioprotection. We propose that the cardioprotective effect of CAPA resulted from its own antioxidant property, which preserves the bioavailable NO in the heart. However, this assumption requires further supported by more direct evidence.

### Effect of chronic CAPA treatment on cardiac dysfunction in diabetes

Animals with STZ-induced diabetes showed reduced resting heart rate and pulse pressure [54]. In our study, the heart rate of diabetic rats was significantly lower than that of control rats during the I/R period, while CAPA treatment reversed the reduction after 45 min of ischemia (Figure 6A). The mean blood pressures of the CAPA-treated groups were higher than those of the other two groups when the hearts were subjected to ischemia for 45 min and higher than those of the STZ-diabetic animals after 60 min of reperfusion (Figure 6B). The pressure preservation effect was in accordance with that seen in our earlier study concluding that CAPA may preserve the vasomotor activity in diabetic animals [31]. The maintenance of blood pressure and heart rate by chronic CAPA treatment may contribute to its protective effect against I/R injury in diabetic animals.

The STZ-induced diabetic animals underwent hemodynamic changes in cardiac function measured by PV loops [55-57]. In our study, the dP/dt max, dP/dt min, ejection fraction, ESPVR, EDPVR, and PRSW values were decreased and the LVEDP value was increased in the STZ-vehicle group; in agreement with the well-established model of STZ-induced diabetic cardiomyopathy. Compared with the STZ-vehicle group, CAPA treatment preserved the basal cardiac function in terms of PRSW, ESPVR, and ejection fraction, but slight preservation of PRSW and stroke work occurred after I/R injury. CAPA treatment did not reverse any parameters of hemodynamic function after reperfusion; however, the ameliorations of these parameters at baseline may contribute to the reduction of infarct size after I/R.

### The underlying mechanisms of CAPA against cardiac dysfunction in diabetes

There are several mechanisms involved in the development of diabetic cardiomyopathy, including increased oxidative stress, and activation of the renin-angiotensin system [58].

In the STZ-induced diabetic rats subjected to I/R injury, hyperglycemia, an independent risk factor, worsens cardiac performance, cell survival, and tissue injury following myocardial I/R via increased oxidant production and reduced antioxidant defenses [2,3,59]. In our study, CAPA ameliorated the I/R injury, but this protective effect disappeared when the –OH functional groups were substituted with methoxyl groups, and the antioxidant activity of CAPA may contribute to the protective effect on the I/R injury and cardiac dysfunction in diabetes.

Intracellular angiotensin II was proven to have a significant role in the pathological process in AT_1a_ receptor-deficient diabetic mice. The inhibition of intracellular angiotensin II level by a renin or angiotensin-converting-enzyme inhibitor prevented the development of cardiac dysfunction in these diabetic mice [60]. AT-1 receptor antagonists attenuate cardiac failure by decreasing cardiac inflammation and normalizing matrix metalloproteinase activity, leading to the alleviation of cardiac fibrosis in STZ-induced diabetic cardiomyopathy [56]. The inhibition of Rho-kinase protects the cerebral barrier from ischemia-evoked injury by modulating endothelial cell oxidative stress and tight junctions [61] and protects the structure and function of cardiac mitochondria from diabetes by attenuating oxidative stress [62]. Acute Rho-kinase inhibition improves coronary dysfunction *in vivo* in the early diabetic microcirculation [63], while long-term Rho-kinase inhibition ameliorates myocardial hypertrophy, apoptosis, fibrosis, and subsequent cardiac remodeling in diabetes [64]. The Rho-associated protein kinase-mediated signaling pathway is known to be involved in the vascular effects of angiotensin II [65], and renin-angiotensin system blockade is beneficial to the cardiovascular system and a known treatment for diabetic cardiomyopathy [66]. In our earlier study, we found that CAPA decreased plasma angiotensin II level in mice with abdominal aortic banding-induced ventricular hypertrophy [34], implicating that the angiotensin II-lowering activity of CAPA may protect the cardiovascular function in diabetes. The level of iNOS is associated with the induction of RhoA expression in the hearts of diabetic rats [67], while CAPA inhibits LPS/IFN-γ-induced iNOS expression [53], implicating that the inhibition of iNOS expression may contribute to its protection against diabetic cardiomyopathy.

Recent evidence shows that an increased infarct size is associated with low levels of myocardial heme oxygenase-1 (HO-1) during I/R in diabetic rats [2,3]. The nuclear factor erythroid 2-related factor 2 (Nrf2) signaling pathway regulates the oxidative stress response, and altered Nrf2 responses may contribute to the observed selective cytotoxicity of electrophilic compounds [68]. Nrf2 is a transcription factor that regulates the expression of many detoxification or antioxidant enzymes [69]. CAPE stimulates *ho-1* gene activity by promoting inactivation of the Nrf2–Keap1 complex, leading to increased Nrf2 binding to the resident *ho-1* AREs, and the induction of HO-1 by CAPE requires Nrf2/ARE pathway activation [70]. In HO-1 activation, CAPA was as effective as CAPE at inducing HO-1 mRNA (nine-fold over vehicle control) as determined by reverse transcription–polymerase chain reaction [71], and CAPA can induce HO-1 mRNA expression in rat primary cultured microglia [53]. These data indicate that CAPA treatment may mitigate infarction in diabetic rats by inducing HO-1 activity.

## Conclusions

STZ-induced diabetic animals suffer from deteriorated cardiac function with increased myocardial infarction following I/R. CAPA can reduce the infarct size after I/R injury, and this study demonstrated that long-term CAPA treatment reversed cardiac dysfunction in diabetic animals. The cardioprotective effect of CAPA may result from its antioxidant activity and the associated preservation of NO-dependent mechanisms. However, the mechanisms of cardiac function preservation in STZ-induced diabetic rats by 4-week CAPA treatment remain to be investigated.

## Abbreviations

+dP/dt: Maximum positive value of the first derivative of the pressure; -dP/dt: Maximum negative value of the first derivative of the pressure; AAR: Area at risk; bpm: Beats per minute; CAPA: Caffeic acid phenethyl amide; CAPE: Caffeic acid phenethyl ester; dmCAPA: Dimethoxyl caffeic acid phenethyl amide; Ea: Arterial volume elastance; EDPVR: End-diastolic pressure volume relationship; ESPVR: End-systolic pressure volume relationship; HO-1: Heme oxygenase-1; I/R: Ischemia-reperfusion; iNOS: Inducible nitric oxide synthase; LAD: Left anterior descending coronary artery; l-NAME: Nω-nitro-l-arginine methyl ester; LPS/IFN-γ: Lipopolysaccharide/interferon-γ; LV: Left ventricle; LVEDP: Left ventricle end-diastolic pressure; LVESP: Left ventricle end-systolic pressure; LVP: Left ventricular pressure; LVV: Left ventricular volume; MDA: malondialdehyde; MPO: Myeloperoxidase; NO: Nitric oxide; NOS: Nitric oxide synthase; Nrf2: Nuclear factor erythroid 2-related factor 2; PEG400: Poly-ethylene glycol 400; PRSW: Preload recruitable stroke work; PV loop: Pressure-volume loop; ROS: Reactive oxygen species; SEM: Standard error of the mean; STZ: Streptozotocin; SV: Stroke volume; SW: Stroke work; TBA: Thiobarbituric acid; TTC: 2,3,5-Triphenyltetrazolium chloride.

## Competing interests

The authors declare that they have no competing interests.

## Authors’ contributions

Participated in research design: YJH, ASL, WPC, WLC and MJS; Conducted experiments: diabetic animal induction: YJH; I/R model: YJH and YKT; PV loop experiments and analysis: YJH, WPC and ASL; Synthesis, purification of CAPA and dmCAPA: HLC and YHK; Performed data analysis: YJH, ASL, WPC, WLC, YKT and MJS; Contributed to the writing of the manuscript: YJH, ASL, WPC, YKT, WLC and MJS; Directed the study and problem solving: MJS. All authors read and approved the final manuscript.
